# Authors’ reply

**Published:** 2010

**Authors:** Varsha Nandedkar, Anil R Joshi, Namrata Kabra, Neha N Deshpande

**Affiliations:** Department of Ophthalmology, Government Medical College, Aurangabad - 431 001, India; 1 Department of Pathology, Government Medical College, Aurangabad - 431 001, India

Dear Editor,

We appreciate the authors[1] of this letter for going through the details of our case report.[2] Fine needle aspiration cytology (FNAC) is contraindicated in a typical case of Retinoblastoma. In this patient, the presentation was an intraocular tumor with vitreous hemorrhage. Considering the age and presentation our differentials were amelanotic melanoma, leukemia, lymphoma, and metastatic tumor. FNAC was the least invasive diagnostic procedure, especially in the third trimester. Therefore, she was advised FNAC, but the patient was lost to follow-up, and returned three months after delivery.

The patient’s categorization was done after enucleation and immunohistochemistry. Till then she was treated as small cell neoplasia. Neo-adjuvent chemotherapy without a confirmed diagnosis, moreover, in the third trimester, was difficult. Due to this limitation she was advised FNAC. When diagnosis from the enucleated eye helped us classify this patient in group E, we went for high dose chemotherapy with radiotherapy as the line of treatment after enucleation.
